# Modeling ameloblast-matrix interactions using 3D cell culture

**DOI:** 10.3389/fphys.2022.1069519

**Published:** 2022-12-01

**Authors:** Gayathri Visakan, Rucha Arun Bapat, Jingtan Su, Janet Moradian-Oldak

**Affiliations:** Center for Craniofacial Molecular Biology, Herman Ostrow School of Dentistry, University of Southern California, Los Angeles, CA, United States

**Keywords:** ameloblasts, ameloblastin, amelogenin, polarization, cell morphology, Geltrex^®^, 3D cell culture

## Abstract

The distinct morphology adopted by ameloblasts during amelogenesis is highly stage specific and involved intimately with the development of a hierarchical enamel microstructure. The molecular mechanisms that govern the development of an elongated and polarized secretory ameloblast morphology and the potential roles played by the enamel matrix proteins in this process are not fully understood. Thus far, the *in vitro* models that have been developed to mimic these early cell-matrix interactions have either been unable to demonstrate direct morphological change or have failed to adapt across ameloblast cell lines. Here, we use a recently established 3D cell culture model to examine the interactions between HAT-7 cells and the major enamel matrix proteins, amelogenin and ameloblastin. We demonstrate that HAT-7 cells selectively respond to functional EMPs in culture by forming clusters of tall cells. Aspect ratio measurements from three-dimensional reconstructions reveal that cell elongation is 5-times greater in the presence of EMPs when compared with controls. Using confocal laser scanning microscopy, we observe that these clusters are polarized with asymmetrical distributions of Par-3 and claudin-1 proteins. The behavior of HAT-7 cells in 3D culture with EMPs is comparable with that of ALC and LS-8 cells. The fact that the 3D model presented here is tunable with respect to gel substrate composition and ameloblast cell type highlights the overall usefulness of this model in studying ameloblast cell morphology *in vitro*.

## Introduction

Enamel’s unique mechanical strength stems from the hierarchical arrangement of hydroxyapatite crystals into prisms with interprismatic mineral (Boyde, 1997; Chai et al., 2009). Although mature enamel is acellular, the generation of a prismatic enamel microstructure has been linked to features of ameloblast morphology during the early stages of amelogenesis - like the Tomes’ processes. During enamel development, ameloblasts undergo a complicated set of movements with each one contributing to unique structural features in mature enamel’s final microstructure. Ameloblasts move away from the developing enamel matrix, and adjacent sheets of ameloblasts move relative to one another resulting in a decussating pattern of enamel rods in higher mammals (Boyde, 1964; Boyde, 1997). However, the mechanisms underlying the formation of this unique, highly specialized ameloblast morphology are not fully understood.

The roles of enamel matrix proteins in amelogenesis - either individually or corporately are constantly evolving (Hatakeyama et al., 2009; Moradian-Oldak, 2012; Mazumder et al., 2016; Lacruz et al., 2017; Bapat et al., 2020; Moradian-Oldak and George, 2021). Genetically engineered mouse models have shown that, in addition to affecting enamel mineralization, components of the extracellular milieu like matrix proteins (ameloblastin and enamelin) and proteinases (MMP20) may influence ameloblast cell behavior (Fukumoto et al., 2005; Guan and Bartlett, 2013; Hu et al., 2014; Shin et al., 2018). The truncation of ameloblastin (removal of exons 5 and 6) results in the loss of secretory ameloblast elongation and polarization with a resultant de-differentiation into an inner enamel epithelium-like stage (Fukumoto et al., 2004). MMP20 overexpression disrupts the coordinated ameloblast movements, resulting in an abnormal migration of the ameloblasts into the papillary layers with a concomitant loss of ameloblast cell polarity (Shin et al., 2018). Additionally, MMP20 has been shown to regulate ameloblast movement through Wnt- and beta-catenin-mediated pathways by catalyzing the cleavage of ameloblast cell surface cadherins (Bartlett et al., 2011; Guan et al., 2016).

Reliable *in vitro* 3D cell culture models have proven invaluable for examining the interactions between ameloblasts and the enamel extracellular matrix (He et al., 2010; Földes et al., 2021). *In vitro* and cell culture systems provide for precise control over experimental conditions and the ability to isolate and examine the role of individual components in the matrix. When primary human enamel epithelial cells are co-cultured with dental pulp cells in a 3D Matrigel, they form spheroidal structures, increasing both their size and the expression levels of integrins (Li et al., 2006; He et al., 2010). Since ameloblasts are lost after amelogenesis, several ameloblast cell lines have been developed that are immortalized, either spontaneously (Nakata et al., 2003) or by viral transfection (Chen et al., 1992). These ameloblast cell lines exhibit characteristic and distinct phenotypes in culture and can represent different ameloblast stages (Sarkar et al., 2014). Using bioreactor-based 3D embedded cultures of HAT-7 cells, it was shown that HAT-7 cells display immunoreactivity to amelogenin and actin when co-cultured with dental pulp cells and in the presence of enamel matrix proteins (Pandya et al., 2021). The differentiation of cells into an ameloblast-like state in 3D culture is often demonstrated with differential gene expression patterns (Jiang et al., 2018). Given the pivotal roles played by ameloblast morphology in patterning the overall microstructure of enamel, ameloblast cell responses need to be methodically characterized, particularly the morphological changes occurring in response to the extracellular matrix composition.

We recently established a 3D cell culture model that is adaptable across gel substrates and allows for the quantification of changes in cell morphology and polarization in response to the matrix composition (Visakan et al., 2022a; Visakan et al., 2022b). Here, our optimized 3D model will be used to culture HAT-7 cells, which are often the cell line of choice for examining functional cell polarization (Bori et al., 2016). Using a systematic protocol for assessing changes in cell morphology by means of cell aspect ratio measurements and polarization by confocal microscopy, we observed that the addition of recombinant amelogenin (AMEL) and ameloblastin (AMBN) to the gel resulted in the formation of elongated and polarized HAT-7 cell clusters. These changes in HAT-7 cells are comparable to those of ALC and LS-8 cells, which have been previously studied (Visakan et al., 2022a). Since the composition of the 3D gel matrix is tunable, this model also allowed us to tailor the cell microenvironment using a combination of proteins or their proteolytic cleavage fragments as shown here with the recombinant AMBN 17 kDa. The proteolytic processing of ameloblastin by MMP-20 results in the production of three N-terminal cleavage fragments that weigh 17, 15 and 13 kDa (Iwata et al., 2007; Chun et al., 2010). Among these N-terminal cleavage fragments, the 17 kDa fragment predominates and exhibits potential regenerative properties when used on artificial periodontal defects (Fukae et al., 2006). We also examined the effect of ameloblastin lacking a potential cell binding domain (Su et al., 2020) on HAT-7 cells, suggesting that this system can be expanded to model disorders of enamel development.

## Methods

### Recombinant protein expression and purification

Recombinant amelogenin and ameloblastin (AMEL, AMBN, AMBNΔ5) were expressed and purified based on published protocols and as described in the Supplementary Material (Su et al., 2019a; Bapat et al., 2020).

### Recombinant 17 kDa ameloblastin cleavage fragment expression, purification and characterization

To purify the 17 kDa fragment of ameloblastin, the full-length Ambn pET32a plasmid was first purified using the Monarch Miniprep kit (New England Biolabs). The 17 kDa fragment within the plasmid was selectively amplified using forward (ata​tat​gga​tcc​gtg​ccg​gca​ttt​cct​caa​c) and reverse primers (ttt​ttt​ctc​gag​tca​acg​ggc​gat​ctg​gaa​c). The PCR product and new pET32a plasmids (EMD Millipore) were digested using BamHI and XhoI enzymes (New England Biolabs). Then, they were purified using a QIAgen gel extraction kit and ligated. The ligated plasmid was transferred into Dh5α competent *E. coli* using the heat shock technique. Bacterial colonies were screened for successful transformation using ampicillin agar plates. The 17 kDa Ambn plasmid from selected colonies was amplified, purified, and sequenced. 17kDa Ambn was expressed in BL21 *E. coli,* and the protein was purified using the previously published protocol for full-length Ambn and characterized as described in the Supplementary Material.

### HAT-7 characterization in 2D cell culture

HAT-7 cells were obtained as a gift from Prof. Hidemitsu Harada (Tokyo). HAT-7 cells were cultured following standard protocols (Kawano et al., 2002). Briefly, cells were cultured in 13 mm culture dishes (Corning) with low glucose Dulbecco’s Modified Eagle Medium (DMEM) (Gibco, Thermo) supplemented with 10% FBS (Thermo) and 1% penicillin/streptomycin (Thermo). This supplemented DMEM is referred to as cell culture media. Cultures were maintained under 5% CO_2_ at 37 °C until they achieved 80% or greater confluence. HAT-7 cells were characterized using immunofluorescence staining for amelogenin and ameloblastin proteins.

### 3D cell culture and aspect ratio measurement

**FIGURE 1:**
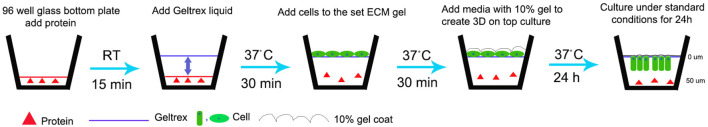
Schematic representation of modified 3D-on-top culture method (side view). Red triangle represents proteins-test (AMEL, AMBN, AMBN 17kDa, AMBN ∆5) and control (BSA, heat denatured AMBN). Green oval and rectangle represent HAT-7 cells. Solid line- GFR Geltrex after polymerization and scalloped line denotes 10% gel overlaid on cells. 0 and 50um markings are used to indicate the Z position on the surface (0 um) and within the gel (50 um) after 24 h resulting in elongation of the cells as shown in Figures 2D,E and Figure 4.

A previously developed, optimized 3D-on-top type culture technique for the culture of ameloblast cell lines was used (Lee et al., 2007; Visakan et al., 2022a; Visakan et al., 2022b). Briefly, pre-chilled glass-bottomed 96-well plates (Mattek) were coated with 20 ug/ml test (AMBN, AMEL, AMBN Δ5, AMBN 17 kDa) and control (heat denatured AMBN, BSA and PBS) proteins. Ice-cold, growth-factor-reduced Geltrex (GFR Geltrex; Thermo) was overlaid on coated plates and incubated at 37 °C for 30 min. HAT-7 cells were detached from the culture dishes at 80% or greater confluence and were centrifuged at 200 x g for 5 min to obtain a soft pellet. The cells were resuspended in cell culture media and inoculated atop set GFR Geltrex gels to create a 3D-on-top-type culture (Figure 1). These 3D gels were incubated at 37 °C in a standard cell culture incubator for 24–72 h. At the end of the experiment, the cells were fixed with 25% glutaraldehyde for 15 min at room temperature. Cells in the 3D gel were labeled with 1:1000 DiD and 1:1000 DAPI and were visualized using Keyence BZX810 with objective PlanApo λ NA 0.75. Sequential Z stacks were recorded with a pitch of 0.4 μm. Z stacks were reconstructed using the Keyence Image Viewer (software version 1.1.1.8), and the 3D measure-tool was used to record the cell width along the XY plane and cell height along the *Z* axis. These values were tabulated, and the aspect ratio was calculated using the following formula: $Aspect\;ratio=\frac{Cell\;height\;\left(Z\right)}{Cell\;width\;\left(XY\right)}$ . Repeated measurements of the aspect ratio were recorded to analyze for statistical significance, and the experiments were repeated three times.

### Immunofluorescence labelling

**FIGURE 2:**
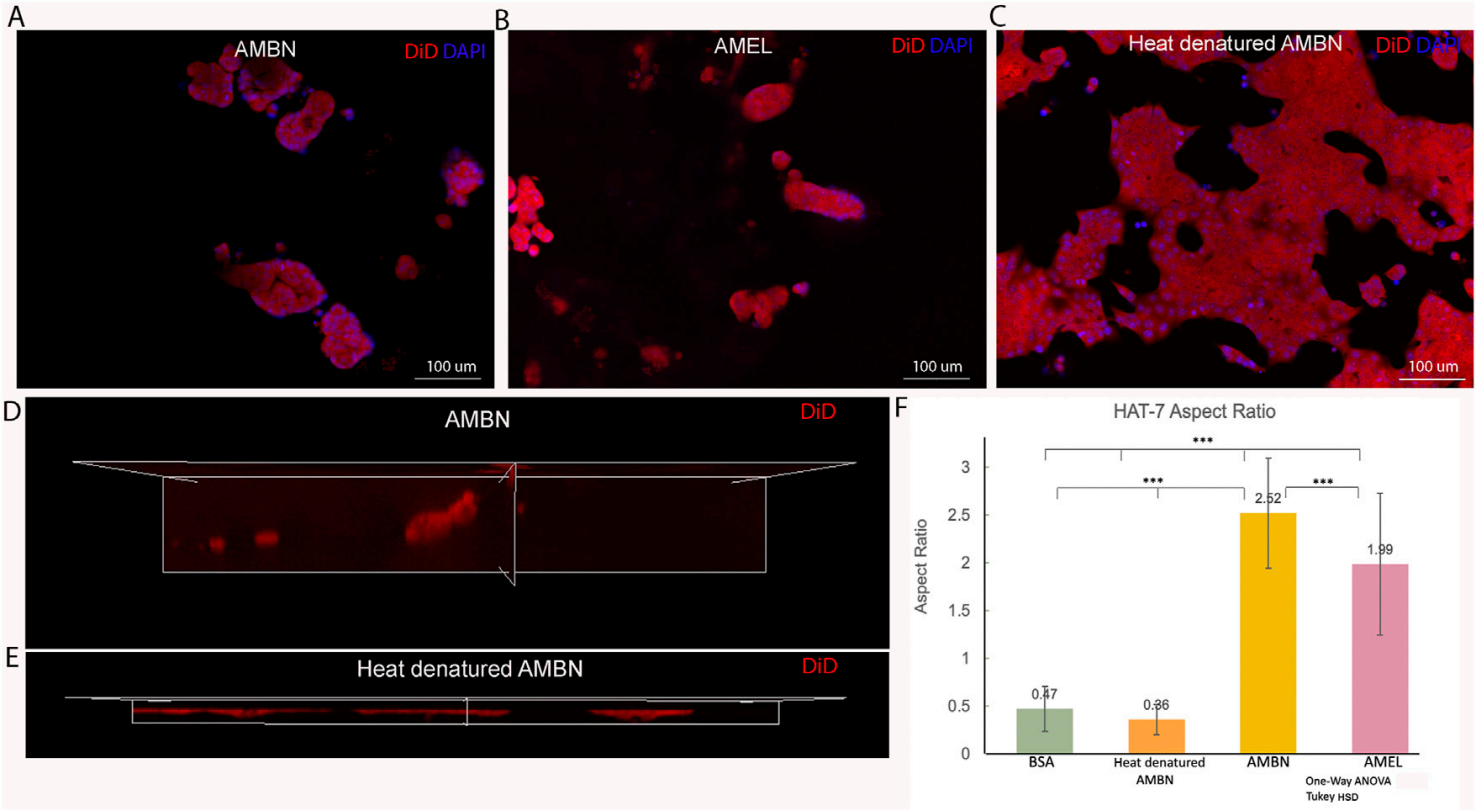
HAT-7 cells in modified 3D-on-top culture using growth-factor reduced Geltrex; 24h. **(A**–**C)**. 2D surface images (top view, XY plane) of HAT-7 cells in the presence of AMBN, AMEL and heat denatured AMBN (negative control) respectively. Cell membrane labeled with DiD (red) and nucleus with DAPI (blue). HAT-7 cells organize into distinct cell clusters in **(A)** and **(B)** unlike in the control **(C)** where the cells are spread along the XY plane. **(D**, **E)**. 3D reconstruction and axial view (side view, XZ plane) of representative samples from AMBN and heat denatured AMBN groups respectively. Cell outline as visualized by DiD reveals elongated, tall cells in **(D)** unlike the planar, flat cells with **(E)**. **(F)** Aspect ratio measurements of HAT-7 cells from test and control groups. HAT-7 cells are statistically significantly elongated in the presence of AMEL and AMBN. *p < 0.05; **p < 0.01; ***p < 0.001.

All steps were carried out in the dark at room temperature. Immunofluorescence labeling of whole cultures was carried out without the extraction of cells from the 3D gel (Lee et al., 2007). Cells in 3D that were fixed with glutaraldehyde were permeabilized with 0.1% Triton-X-100 for 5 min at room temperature. Then, the cells were incubated with 10% serum from the species of secondary antibody for 30 min at room temperature and were labelled with primary antibodies against Par-3, Actin and claudin-1. Fluorescently conjugated secondary antibodies (Jackson Immuno) were used for immunodetection for 1 h at room temperature. For characterization of HAT-7 cells, anti-amelogenin and anti-ameloblastin antibodies were used. A 2D monolayer of cells was fixed using 4% paraformaldehyde, was permeabilized using 0.1% Triton-X-100, and was labeled following standard protocols for immunofluorescence. Antibody dilutions for both 3D and 2D are provided in Supplementary Table S1. All images were created with confocal microscopy as described, except the image in Figure 2 and Supplementary Figure S2 which was made using an inverted fluorescence microscope (Keyence BZX810, Keyence, United States).

### Confocal imaging

Confocal imaging was carried out using the Lecia Stellaris 5 confocal microscope with an oil immersion objective HCX PL APO CS 63 x (NA 1.4). Sequential Z stacks were recorded with an optical pitch of 0.5 µm. Alexa Fluor 488 was detected at 502–552 nm (excitation at 488 nm), DiD was detected at 645–695 nm (excitation at 633 nm), and Alexa Fluor 647 was detected at 650–695 nm (excitation at 647 nm). Z stacks were 3D reconstructed and viewed using the LAS-X version 1.8.1.13759.

### Statistical analysis

Data from aspect ratio measurements was analyzed with Microsoft Excel (version 16.66.1) using One-way ANOVA, independent Student’s *t*-test and Tukey HSD. *p* values less than 0.05 were considered as statistically significant. All experiments were performed in triplicates. For all experiments 90 cells in total were examined.

## Results

### 3D culture to analyze morphology and aspect ratios in HAT-7 cells in response to AMEL and AMBN

Confluent 2D monolayers of HAT-7 cells were characterized by immunofluorescence staining for amelogenin and ameloblastin. We observed the expression of both proteins intracellularly using confocal microscopy, confirming the ameloblast characteristics of the HAT-7 cells (Supplementary Figure S1). HAT-7 cells that were cultured in the presence of enamel matrix proteins in 3D culture displayed characteristic differences in their morphology when compared with controls at the end of 24 h (Figure 2). HAT-7 cells organized into clusters in the presence of AMBN and AMEL as visualized with cell membrane labeling using DiD (Figures 2A,B). This morphology adopted by the HAT-7 cells contrasts strikingly with the controls (gel alone, BSA and heat denatured ameloblastin) where cells-maintained cell-cell contacts and were spread along the XY plane (Figure 2C). 3D reconstruction from sequential Z stacks revealed that the HAT-7 clusters in the presence of AMEL and AMBN were composed of tall cells that preferentially elongated along the *Z* axis (Figure 2D). Meanwhile, in the controls, the cells appeared planar without any significant height (Figure 2E). To quantify the cell elongation that occurs in the presence of enamel matrix proteins (EMPs), the aspect ratio of the individual cells was recorded. Repeated measurements of the cell aspect ratio revealed that, after 24 h of 3D culture, HAT-7 cells elongated 5.42 times greater with EMPs when compared to the controls (i.e., in the presence of BSA and heat denatured AMBN). This increase in the aspect ratio was statistically significantly different between all groups (*n* = 90; *p* < 0.001) with the greatest cell elongation achieved in the presence of AMBN (*n* = 90; *p* < 0.001) (Figure 2F). This pattern of cell behavior was consistent at the end of 72 h of culture (Supplementary Figure S2).

### Morphology and aspect ratios in HAT-7 cells in response to 17 kDa AMBN and AMBN Δ5

**FIGURE 3:**
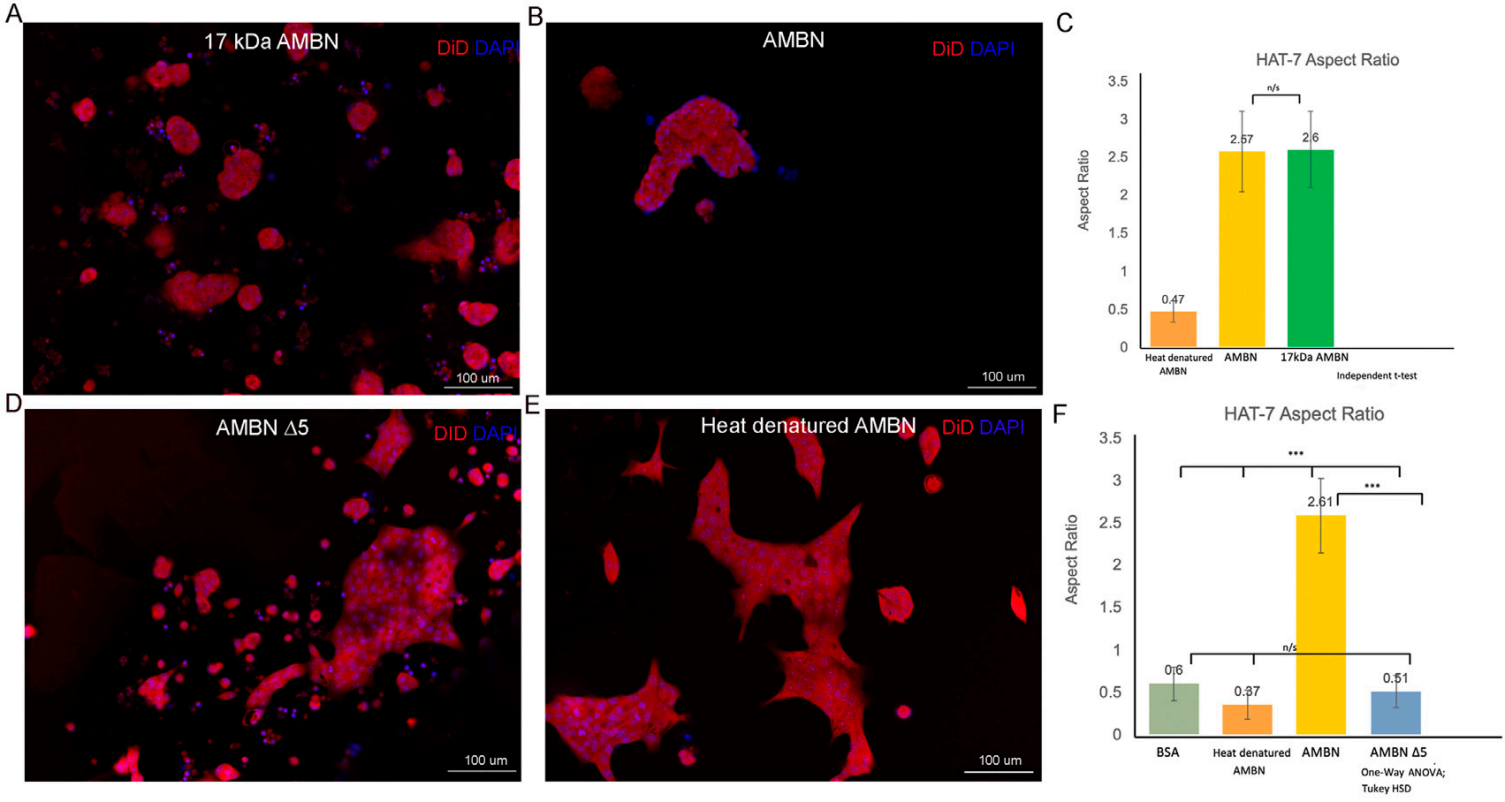
HAT-7 cells cultured with AMBN 17 kDa fragment and AMBN ∆5 mutant. **(A**,**B)**. XY surface images of HAT-7 cells cultured in the presence of AMBN 17 kDa fragment and wild-type full-length AMBN respectively. HAT-7 cells organized into distinct clusters of cells in both groups as visualized using DiD (red) and DAPI (blue). **(C)**. Aspect ratio measurements of HAT-7 cells cultured with 17 kDa fragment, positive and negative controls revealing statistically comparable HAT-7 cell elongation in AMBN 17 kDa cleavage fragment and positive control (full-length AMBN); *p* > 0.05. **(D**,**E)**. XY surface images of HAT-7 cells cultured with mutant AMBN ∆5, and heat denatured AMBN respectively. **(F)**. Aspect ratio measurements of HAT-7 cells in the presence of AMBN **∆**5. Removal of exon 5 encoded sequence resulted in HAT-7 cells remaining planar with aspect ratio values statistically significantly lower than wild-type AMBN *p* < 0.001. AMBN ∆5 aspect ratios are comparable with those of negative controls *p* > 0.05. *p < 0.05; **p < 0.01; ***p < 0.001.

Recent work has identified the presence of a highly conserved amphipathic helix (AH) forming, cell binding motif within the exon 5 region of AMBN (Su et al., 2019b; Su et al., 2020). To verify the function of the N-terminal domain identified within exon 5 of *Ambn,* the 3D culture model was used to examine the effect of 17 kDa AMBN (a proteolytic fragment contains the AH domain) and AMBN Δ5 (a mutant lacks the AH domain) on HAT-7 morphology. The theoretical molecular mass of AMBN 17 kDa was calculated using ProtParam and was found to be 17,990.53 Da. SDS-PAGE gels revealed that the protein migrated at an apparent molecular weight higher than the expected theoretical value (Supplementary Figure S3A). Mass spectra (ESI) of the band around 20 kDa in SDS-PAGE showed that the molecular mass of the protein was 17,991 Da, close to the theoretical value of 17,990.53 Da, suggesting that the purified protein was the AMBN 17 kDa fragment (Supplementary Figure S3B). The difference in the theoretical and experimental mass of the protein was 0.47 Da, suggesting that the protein identified in the peak was intact. The amino acid sequence of the 17 kDa cleavage fragment is shown in Supplementary Figure S3C. When cultured with the recombinant AMBN 17 kDa cleavage fragment, HAT-7 cells responded similarly to full-length ameloblastin. At comparable molar concentrations of both proteins (0.48 μM), the 17 kDa fragment was sufficient to recapitulate the cell clustering and elongation achieved with full-length AMBN (Figures 3A,B). Measurements of the aspect ratio confirmed this, where no statistically significant difference was observed between the 17 kDa fragment and the full-length AMBN (*p* > 0.05) (Figure 3C). The 3D model was then used to observe how the removal of highly conserved key domains from AMBN affected HAT-7 morphology. Culturing HAT-7 cells with AMBN Δ5 lacking the exon 5 encoded region resulted in a complete reversal of the cells’ behavior in 3D. HAT-7 cells failed to cluster and, instead, appeared distinct like in the negative controls (Figures 3D,E). Aspect ratio measurements confirmed the lack of cell elongation in the AMBN Δ5 mutant when compared with the wild-type AMBN (*p* < 0.001) (Figure 3F).

### Asymmetric distribution of Par-3 and claudin-1 upon HAT-7 polarization with AMBN

**FIGURE 4:**
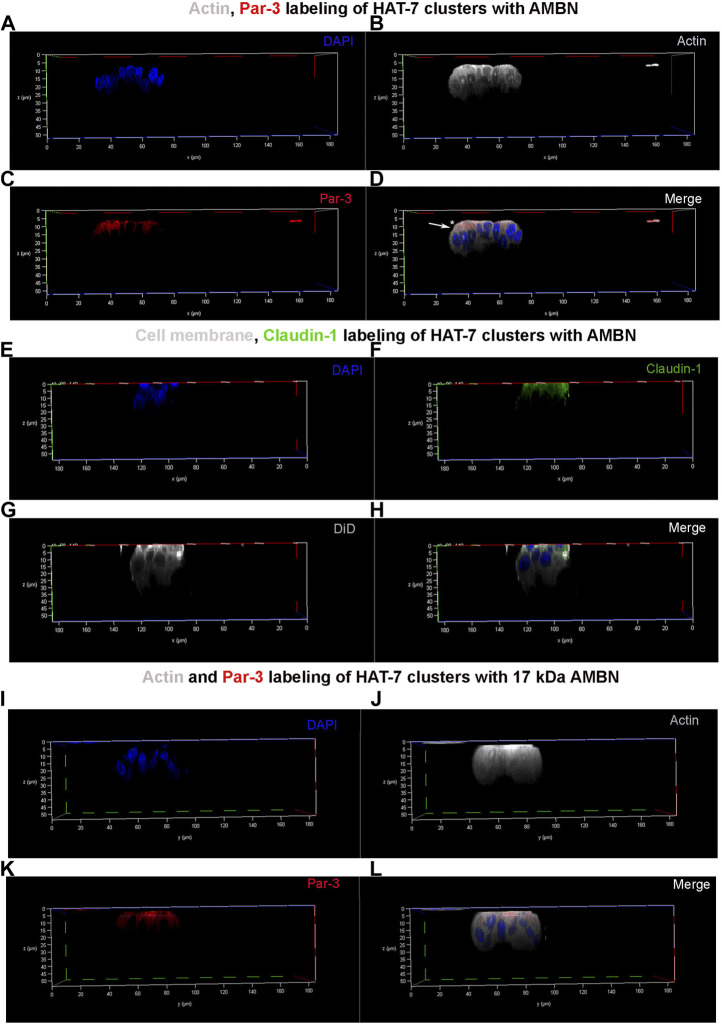
Par-3 and Claudin-1 polarization in HAT-7 clusters in a 3D axial view. **(A**–**C)** Individual channels for nucleus (DAPI), actin (grey pseudo color) and Par-3 (red) distribution respectively in HAT-7 cells cultured with AMBN. **(D)** Merged image of all three channels revealing a uniform symmetrical labeling of actin with an asymmetrical and polarized distribution of Par-3 (white arrow). **(E**–**G)** Individual channels for nucleus (blue), claudin-1 (green) and DiD (grey pseudo color) respectively in HAT-7 cells cultured with ameloblastin. **(H)** Merged images of all three channels revealing selective claudin-1 labeling within the pole of the cells basal to nucleus. DiD labeling is uniform throughout the entire outline of the cells. **(I**–**K)** Individual channels for nucleus (DAPI), actin (grey pseudo color) and Par-3 (red) distribution in HAT-7 cells cultured with AMBN 17 kDa cleavage fragment respectively. **(L)** Merged image of all three channels revealing a uniform symmetrical labeling of actin with an asymmetrical and polarized distribution of Par-3 like the observations in **(D)**.

3D clusters of HAT-7 cells that were elongated in the presence of AMBN were labeled with anti-actin, anti-Par-3, and anti-claudin-1 antibodies (Figure 4). They were then examined under a confocal laser scanning microscope with the 3D reconstruction of sequential Z stacks. Actin labeling (grey pseudo color) confirmed the cell elongation (Supplementary Figure S4A,B) observed earlier with DiD (Figure 2). Actin labeling patterns within individual cells in HAT-7 clusters (formed in the presence of AMBN) were uniform and symmetrical with signals being detected throughout the entire outline of the cells (Figure 4B). Cell polarity protein Par-3 (red) localization, however, was polarized with signals being restricted to the cell membrane basal to nucleus (Figure 4C). Additionally, colocalization of actin and Par-3 was restricted to one pole of the cell (white arrows in Figure 4D). XY plane images acquired at two different Z depths from the surface of the cells revealed that, at higher Z depths, only actin signals were detectable, which confirms the polarized distribution of Par-3 (Supplementary Figures S5A–F). Like Par-3, tight junction protein claudin-1 (green) was also asymmetrical in its distribution compared to that of cell membrane label DiD (grey pseudo color) with its localization being restricted basal to nucleus (Figures 4E–H). The planar cells in the control did not display any polarization in the distribution of Par-3 (Supplementary Figure S6). The HAT-7 clusters formed in the presence of 17 kDa AMBN were also polarized with the Par-3 localization patterns being comparable with that of the full-length AMBN (Figures 4I–L).

## Discussion

It has been proposed that ameloblast morphology is directly or indirectly related to the development of an overall prismatic enamel architecture (Boyde, 1964, Boyde, 1997; Nanci, 2017). Ameloblast elongation and polarization were severely impacted in genetically engineered mouse models lacking functional ameloblastin or enamelin (Fukumoto et al., 2004; Hu et al., 2014), resulting in a lack of true enamel. The severe dysmorphology in genetically engineered animal models makes it challenging to observe and examine the molecular mechanisms that govern the formation of this highly specialized ameloblast morphology, thereby necessitating the need for *in vitro* models.

Here, we demonstrate that the changes in cell morphology and polarization upon addition of enamel matrix proteins can be modeled using a recently-established, *in-vitro* 3D cell culture (Visakan et al., 2022a). This model enabled such differential cell responses to be readily visualized and quantified in a systematic manner in three different ameloblast-like cell lines (i.e., ALC, LS-8 and HAT-7). Using immunofluorescent staining and aspect ratio measurements, we observed that HAT-7 cells exhibited a preferential elongation along the *Z* axis, resulting in the formation of clusters of tall cells when cultured in the presence of AMBN or AMEL. Formation of these tall cells in the presence of the 17 kDa AMBN suggests a potential *in vivo* significance of this response, considering that the 17 kDa cleavage fragment persists in the prism sheaths of the developing enamel matrix (Uchida et al., 1991).

Loss of function of AMBN in cell elongation and polarization was further demonstrated using recombinant mutant AMBN protein lacking the exon 5 encoded region (AMBN Δ5). This mutant lacked the ability to induce cell clustering or elongation in HAT-7 cells. This observation can be the result of loss of AMBN -cell binding, (Su et al., 2020), or AMBN self-assembly (Wald., et al., 2017). AMBN exon 5 encoded region contains the amphipathic helix (AH) cell binding domain that recently was found to be highly conserved among mammals and had a strong evolutionary relationship with enamel prismatic structure (Su et al., 2022). The same region contains the Y/F-x-x-Y/L/F-x-Y/F self-assembly motif, and the disruption of this motif resulted in a perturbed enamel prismatic architecture (Wald et al., 2017). The Y/F-x-x-Y/L/F-x-Y/F motif within exon 5 encoded region also is the site of co-assembly with AMEL (Bapat et al., 2020).

The model can also be used to examine and characterize the polarization status of cells in 3D culture as demonstrated using immunofluorescent labeling of Par-3, claudin-1, and actin proteins. We originally developed this 3D on top culture model to generate non-uniform contact surfaces for cells to mimic the enamel extracellular matrix during development (Visakan et al., 2022a). The developing ameloblasts are in contact with the ECM only along their functional apical membranes, and this is what was modeled in the modified 3D culture system. Culturing HAT-7 cells in 3D-on-top-type cultures reveal a differential cell behavior in the presence of AMEL and AMBN with the HAT-7 cells adopting distinctly different morphologies. Like previous observations with other ameloblast cell lines - ALC and LS-8 cells (Visakan et al., 2022a; Visakan et al., 2022b). HAT-7 cells exhibit preferential elongation along their *Z* axes with aspect ratios 5-times greater than control when amelogenin and ameloblastin were added to the 3D cell culture. In repeated experiments, AMBN was observed to exert a greater influence on HAT-7 elongation than amelogenin did. These morphological changes were specific to the presence of functional enamel matrix proteins, as this behavior could be reversed using heat denatured AMBN.

HAT-7 cells derived from rat molars are functional ameloblast-like cells expressing amelogenin and ameloblastin proteins (Kawano et al., 2002). They are often the cell line of choice for examining functional cell polarization (Bori et al., 2016), the epithelial-mesenchymal interactions during odontogenesis (Matsumoto et al., 2011), and for modeling disease (Földes et al., 2021). When cultured on permeable transwell filters, HAT-7 cells formed polarized epithelial layers as characterized by apical membrane permeability to CO_2_ and basolateral membrane uptake of bicarbonate (Bori et al., 2016). These functional polarized layers also express the tight junctional proteins claudins-1, 4 and 8. In the 3D-on-top culture, we observed that the HAT-7 cells cultured in the presence of AMBN and 17 kDa AMBN exhibited features of structural cell polarization (Figure 4).

Confocal laser scanning microscopy revealed a selective accumulation of polarity protein Par-3 and tight junctional protein claudin-1 along the HAT-7 membrane basal to nucleus position in 3D (Figure 4). Claudin-1 was chosen as a marker for polarization as it has been shown to be highly upregulated in the elongated secretory ameloblasts (Zhang et al., 2019). Furthermore, in fully polarized mouse ameloblasts, it has been observed that Par-3 localized along the functional base and claudin-1 along the functional apical membrane (Inai et al., 2008). 3D reconstruction of Z stacks permits the precise localization of claudin-1 and Par-3 within the overall outline of the cell visualized with actin. Par-3 and claudin-1 labeling patterns in 3D culture of ALC (Visakan et al., 2022a) and HAT-7 cells (Figure 4) contrasts with what is observed *in vivo*. In 3D cultures, both proteins were observed to be localizing along the same pole of the cell. This discrepancy could be due to the differences in the height of the secretory stage ameloblasts in fully developed enamel organs compared to the individual cells in 3D culture. We have recently reported that the addition of functional AMBN to LS-8 cells in culture resulted in an upregulation of planar cell polarity protein Vangl2 (2.8 times) and cell polarity protein Par-3 (3.8 times) when compared to controls (heat denatured AMBN and AMBN Δ5) (Su et al., 2022). Vangl2 planar cell polarity protein is highly expressed particularly in the Tomes’ processes of secretory stage ameloblasts (Nishikawa and Kawamoto, 2012).

Upon their secretion into the enamel matrix, amelogenin and ameloblastin are rapidly processed into N- and C-terminal cleavage fragments by MMP-20 (Bartlett and Simmer, 1999). An evolutionarily conserved novel amphipathic helix (AH) forming cell binding domain within the exon-5 encoded region of ameloblastin was recently identified (Su et al., 2019b). Using the immunofluorescent localization of peptides, it was observed that the AH domain could be specifically localized to ALC cell processes (Su et al., 2020). Furthermore, in ameloblast cell lines, the gene expression levels of cell polarity and planar cell polarity proteins were upregulated in the presence of ameloblastin (Su et al., 2022). In the present study, given the rapid *in vivo* processing of ameloblastin, the newly expressed recombinant 17 kDa ameloblastin containing the exon 5 encoded region was tested in 3D on HAT-7 cells to examine the potential *in vivo* significance of ameloblastin-cell interactions. All the cell effects that were observed with full-length ameloblastin were observed in the presence of the 17 kDa ameloblastin cleavage fragment as well. HAT-7 cells form clusters of elongated cells with polarized distributions of Par-3 (Figures 3, 4).

In conclusion, 3D cell culture models are increasingly replacing conventional 2D monolayer culture techniques, as they result in physiologically comparable cell responses (Kozlowski et al., 2009; Baker and Chen, 2012; Duval et al., 2017). However, the validity of the model lies in large part in being replicable across cell lines and substrate types. The 3D-on-top model presented here is adaptable across gel substrates (GFR Geltrex, type I collagen and gelatin) and can be used with multiple ameloblast-like cell lines (ALC, LS-8 and HAT-7) (Visakan et al., 2022a; Visakan et al., 2022b). Although the 3D system presented here permits visualization of the cells and the structures they adopt in 3D, they are limited in their localization of the EMPs within the entirety of the gel. Examination of the diffusion gradients within the gel and the effect of the gel’s rheological properties remain outside the scope of this study. The interactions of the ameloblast-like cells with enamel extracellular matrix proteins can be modeled using the *in vitro* 3D cell culture system. Such 3D culture models can be expanded to observe the concerted effects of the various bioactive molecules in the extracellular milieu on the cells, and the knowledge thus gained may be translated into future biomimetic attempts at enamel repair.

## Data Availability

The original contributions presented in the study are included in the article/Supplementary Materials, further inquiries can be directed to the corresponding author.
