# Supplementary material for: Modeling ameloblast-matrix interactions using 3D cell culture
Source: Front Physiol. 2022 Dec 1;13:1069519. doi: 10.3389/fphys.2022.1069519 (PMC9751369; doi:10.3389/fphys.2022.1069519)
Supplement: Supplementary file 1 [file Presentation1.pdf]

Supplementary Material

Modeling ameloblast-matrix interactions using 3D cell culture

Gayathri Visakan, Rucha Arun Bapat, Jingtan Su, Janet Moradian-Oldak*

Center for Craniofacial Molecular Biology, Herman Ostrow School of Dentistry, University of Southern California, Los Angeles, CA, United States.

*All correspondence may be addressed to:

Dr. Janet Moradian-Oldak, Center for Craniofacial Molecular Biology, University of Southern California,

Los Angeles, CA.

Phone: 323-442-1759

Fax: 323-442-2981

Email: joldak@usc.edu

Materials and Methods

Recombinant protein expression and purification

Recombinant full-length mouse amelogenin rM179 was expressed in BL21 *E. coli* cells, precipitated with saturated ammonium sulphate, and purified using reverse phase HPLC. Recombinant full-length mouse ameloblastin and ameloblastin mutant lacking the exon 5 encoded region (AMBN $\Delta 5$) were expressed in BL21 *E. coli* cells. The protein was then affinity purified using Ni-NTA agarose (Qiagen), dialyzed against a 10 kDa MWCO snakeskin dialysis membrane (Thermo), and purified using reverse phase HPLC after removal of the thioredoxin and S-tags using enterokinase (New England Biolabs). Heat denaturation of recombinant mouse ameloblastin was carried out by heating the protein in PBS for 15 minutes at 80 °C in a heating block.

Recombinant 17kDa AMBN expression, purification and characterization

The HPLC system (Varian) equipped with a Reversed Phase C4 column (Phenomenex) was used to purify the 17 kDa AMBN protein. The purified protein was lyophilized at -80 °C overnight and stored in -20 °C. Then, it was dissolved in ultrapure water (Thermo) until use. The protein was characterized using 12% SDS-PAGE gel electrophoresis and intact protein analysis through mass spectrometry (ESI and MALDI). The 17 kDa protein mass analysis was carried out at the Scripps Center for Metabolomics and Mass Spectrometry in San Diego, California, United States.

Mass Spectrometry (ESI and MALDI) performed at Scripps Center for Metabolomics

For ESI analysis, an Agilent PLRP-S 1000Å 5μm was used along with a column guard to protect the column from overloading and from particulates. Autosampler used is Agilent Technologies 1200 series with an Agilent Technologies 6230 TOF LC/MS (Dual AJS ESI ion source). Solvent A is 0.1% formic acid in water and Solvent B is 0.1% formic acid in acetonitrile. ESI data was processed using the Agilent MassHunter BioConfirm softwatre. For MALDI, Bruker Autoflex Max was used. Samples were spotted on a MTP 384 ground steel plate. For most samples, spot 0.5 uL of undiluted sample, then spot 0.5 uL of appropriate matrix was used. In the adjacent well, 0.25 uL of water, 0.25 uL of sample, and 0.5 uL of appropriate matrix (diluted sample by half) were spot. For proteins we use a saturated Sinapinic acid matrix prepared in 50/50 ACN/H2O with ~0.1% TFA. MALDI is included in Intact protein analysis as a quality control measure.

Supplementary Table 1. Antibody dilutions used for immunofluorescent staining of 2D and 3D cell cultures.

**Protein**	**Antibody**	**Host**	**Concentration for IF**
Actin	Abcam – ab-8227	Rabbit	5 ug/ml
Par3	Proteintech-11085-1-AP	Rabbit	1: 400
Claudin-1	Proteintech-28674-1-AP	Rabbit	1: 200
Amelogenin	Abcam- ab-153915	Rabbit	1: 5000
Ameloblastin	R&D systems- AF3026	Goat	1: 5000
Monovalent Fab (anti-rabbit)	JacksonImmuno-AB_2340587	Donkey	30 ug/ml

Supplementary Figures

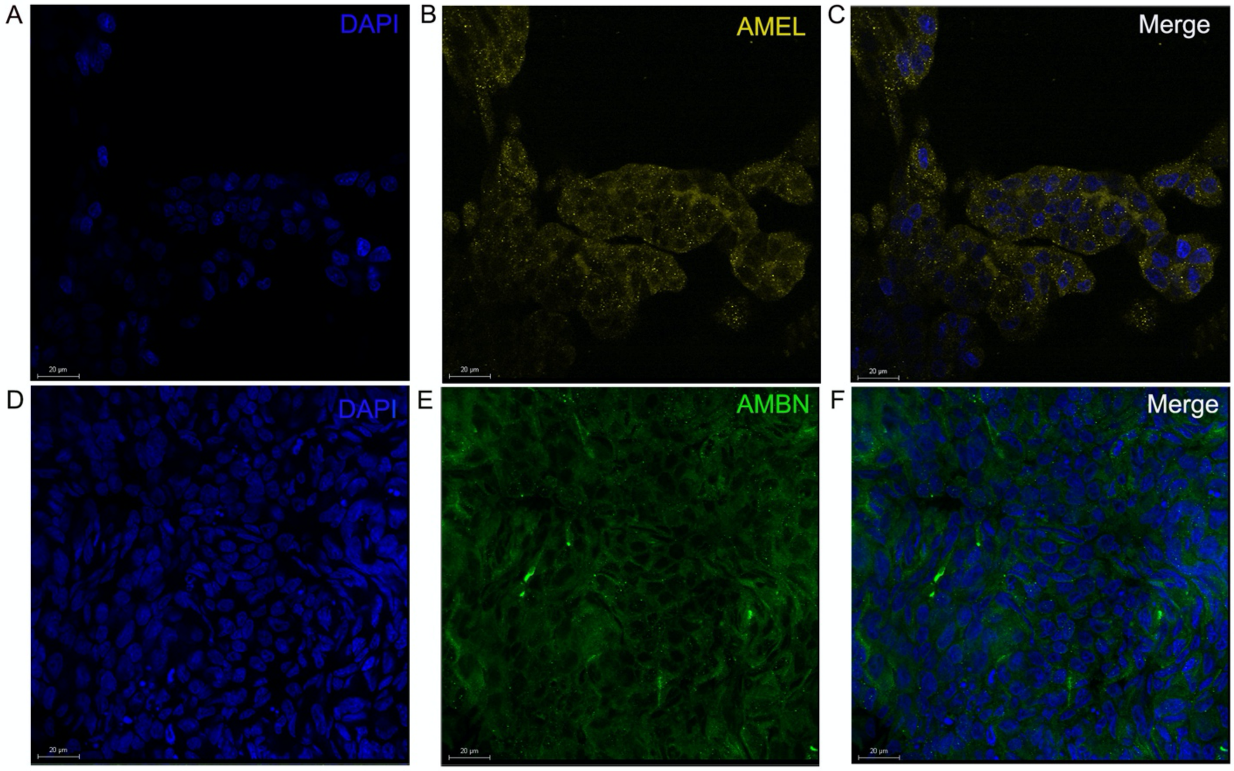

Supplementary Fig 1. Characterization of HAT-7 cells with anti-AMEL and anti-AMBN antibodies. (A-C) confluent HAT-7 cells labeled with amelogenin (yellow) and nucleus (blue). (D-F) confluent HAT-7 monolayers labeled with ameloblastin (green) and nucleus (blue).

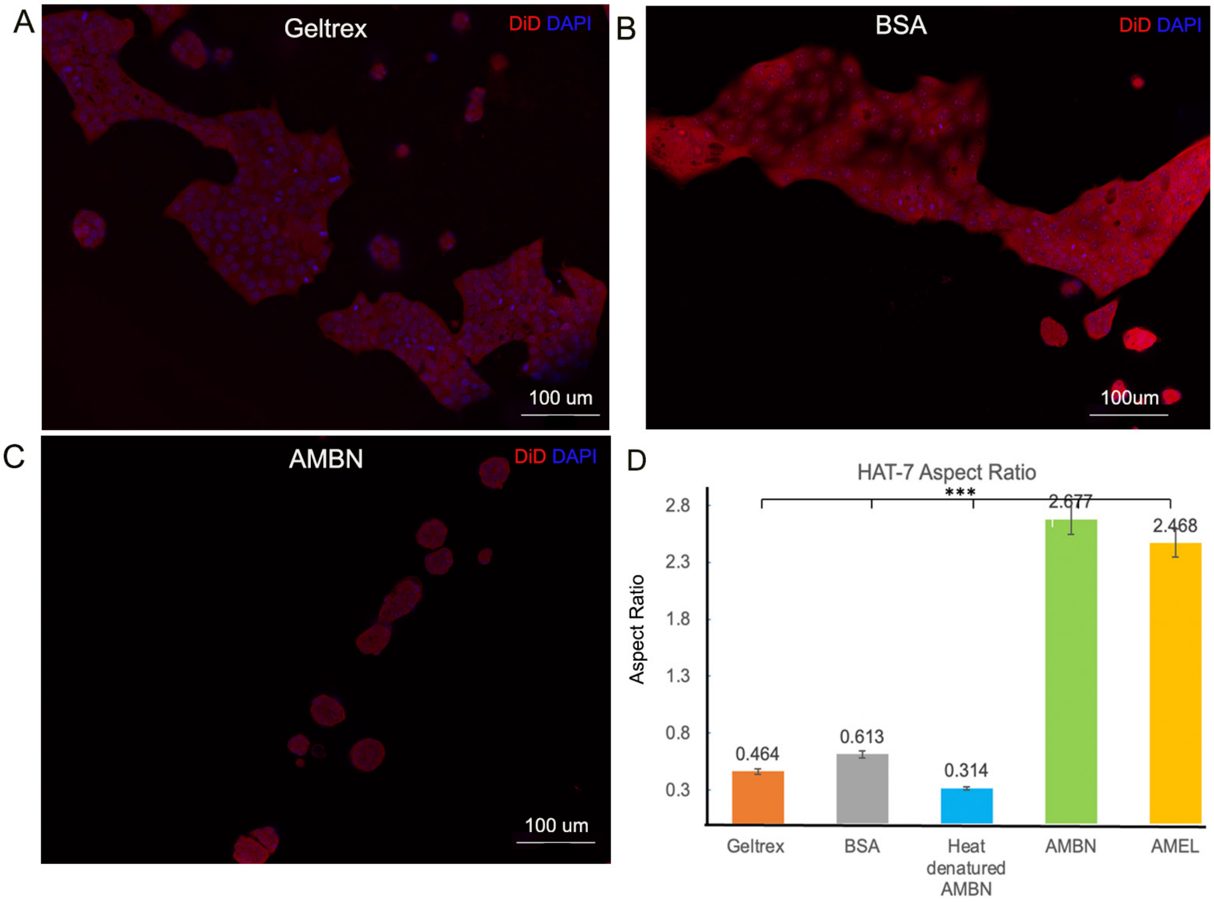

Supplementary Fig 2. 3D culture of HAT-7 cells in growth-factor reduced Geltrex gels, 72h. (A, B) XY surface image of HAT-7 cells in the presence of Geltrex alone and bovine serum albumin respectively. (C) XY surface image of HAT-7 cell clusters formed in the presence of AMBN. Cell membrane labeled with lipophilic dye DiD (red) and nucleus with DAPI (blue). (D) Aspect ratio measurements of HAT-7 cells at the end of 72h of culture. There's a statistically significant difference between the groups tested with HAT-7 cell elongation occurring only in the presence of AMBN and AMEL. *** $p < 0.001$.

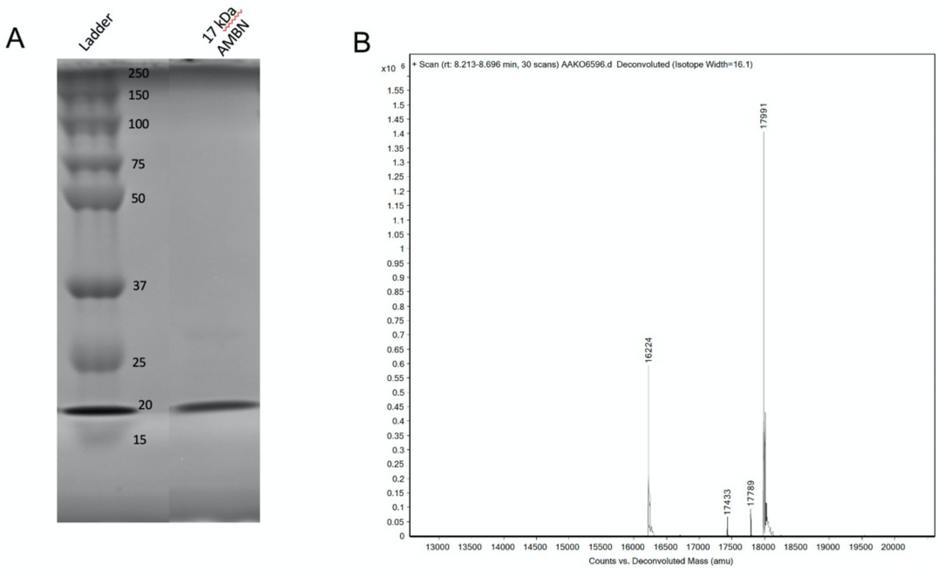

C

```
        10         20         30         40         50         60
AMADIGSVPA FPQQPGAQGM APPGMASLSL ETMRQLGSLQ GLNALSQYSR LGFGKALNSL

        70         80         90        100        110        120
WLHGLLPPHN SFPWIGPREH ETQQPSLQPH QPGLKPFLQP TAATGVQVTP QKPGPQPPMH

       130        140        150        160
PGQLPLQEGE LIAPDEPQVA PSENPPTPEV PIMDFADPQF PTVFQIAR
```

Supplementary Fig 3. Characterization of the recombinant AMBN 17kDa cleavage fragment by 12% SDS-PAGE and Mass Spectrometry (ESI). (A) 12% SDS PAGE gel stained with Coomassie Blue showing the 17kDa fragment migrating close to 20kDa when compared with the molecular standard. (B) ESI spectrum of the protein that migrated at 20kDa in SDS-PAGE revealing a peak at 17991 Da. (C) Schematic representation of the amino acid sequence of the 17kDa fragment.

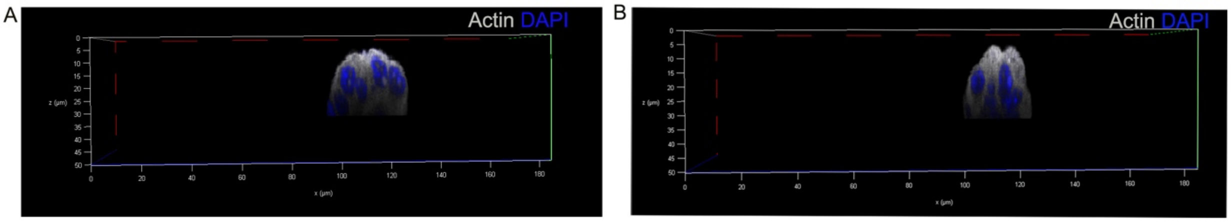

Supplementary Fig 4. Three-dimensional reconstruction of HAT-7 cells cultured in the presence of AMBN. (A, B) Represent two different areas from a representative cluster of HAT-7 cells labeled with anti-Actin antibody (grey pseudo color) and DAPI (blue). Actin labeling revealing a preferential elongation of the HAT-7 cells along the Z axis.

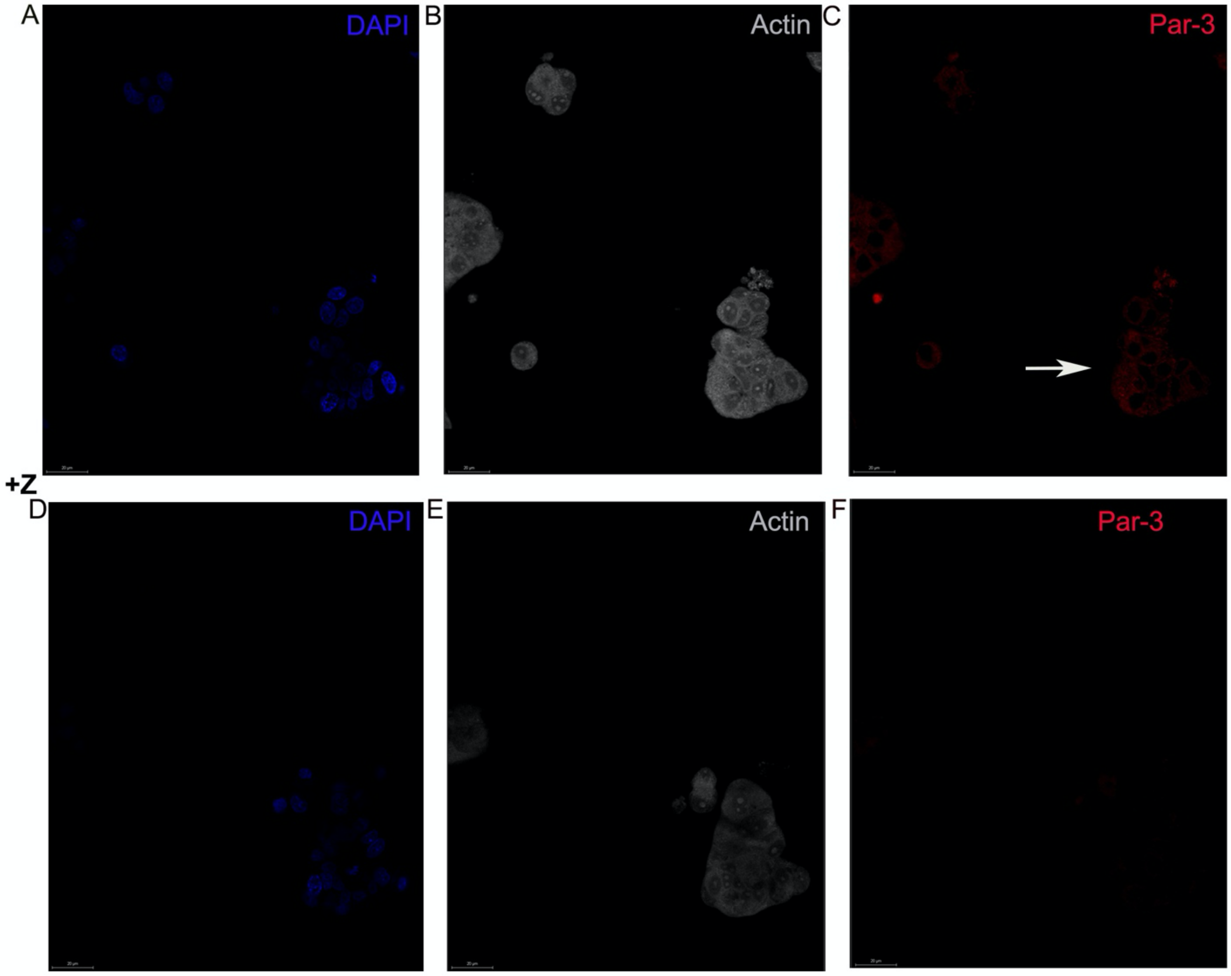

Supplementary Fig 5. 2D orthogonal sections of a representative 3D cluster of HAT-7 cells cultured in the presence of AMBN at two different Z depths from the surface. (A-C) represent individual channels for nucleus (blue, A), actin (grey pseudo-color, B) and Par-3 (red, C). White arrow in C used to indicate presence of Par-3 signal at this Z depth. (D-F) represent individual channels for nucleus (blue, D), actin (grey pseudo-color, E) and Par-3 (red, F) at a greater Z depth from surface. No signal detected for Par-3 channel at this Z depth while actin signals are still detectable.

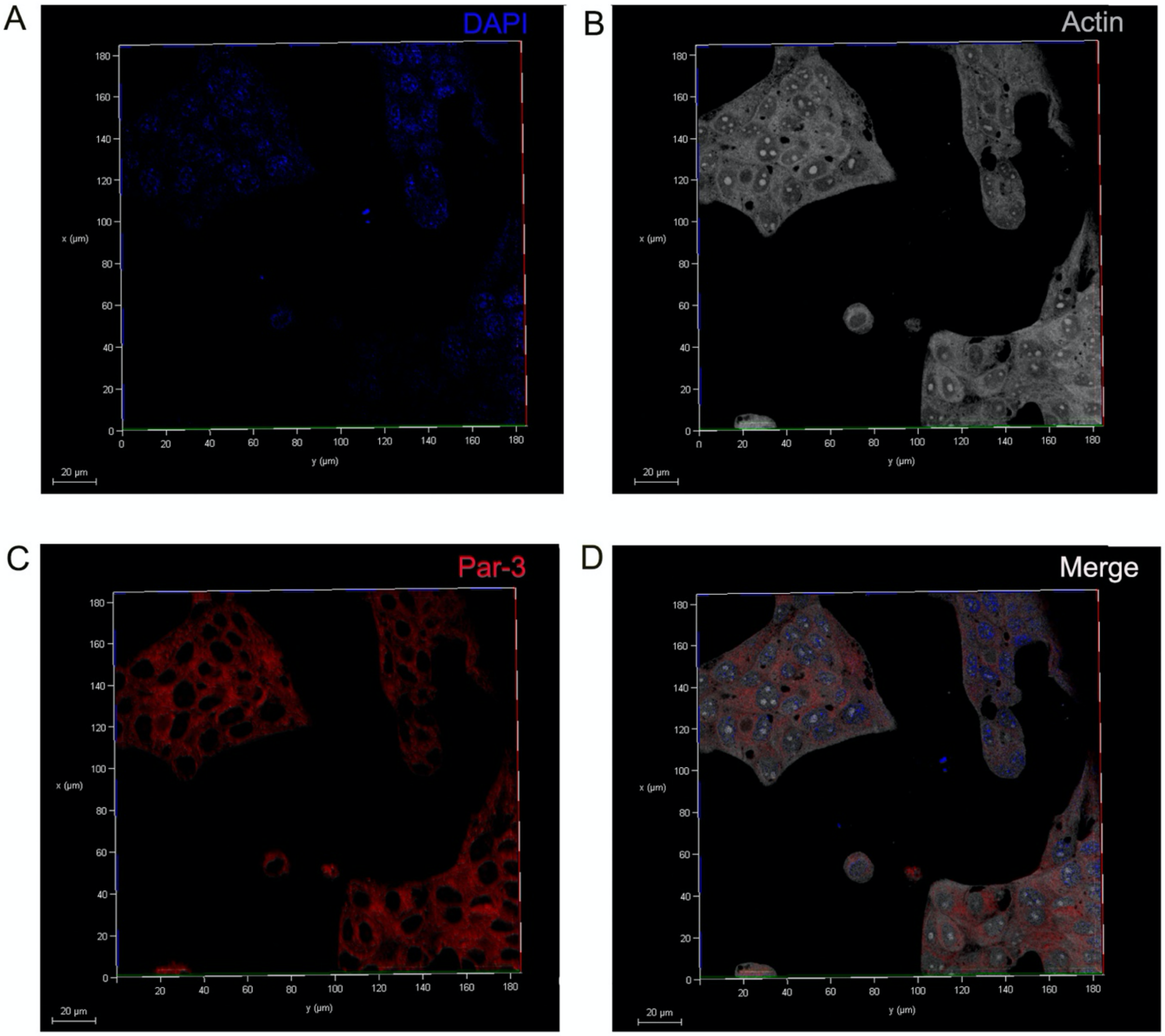

Supplementary Fig 6. Actin and Par-3 labeling in control (heat denatured AMBN). (A-C) represent individual channels for nucleus (blue), actin (grey pseudo-color) and Par-3 (red) respectively. HAT-7 cells are planar without any asymmetry in the distribution patterns of Par-3 within the actin labeled cells. (D) represents a merged image containing all three channels.

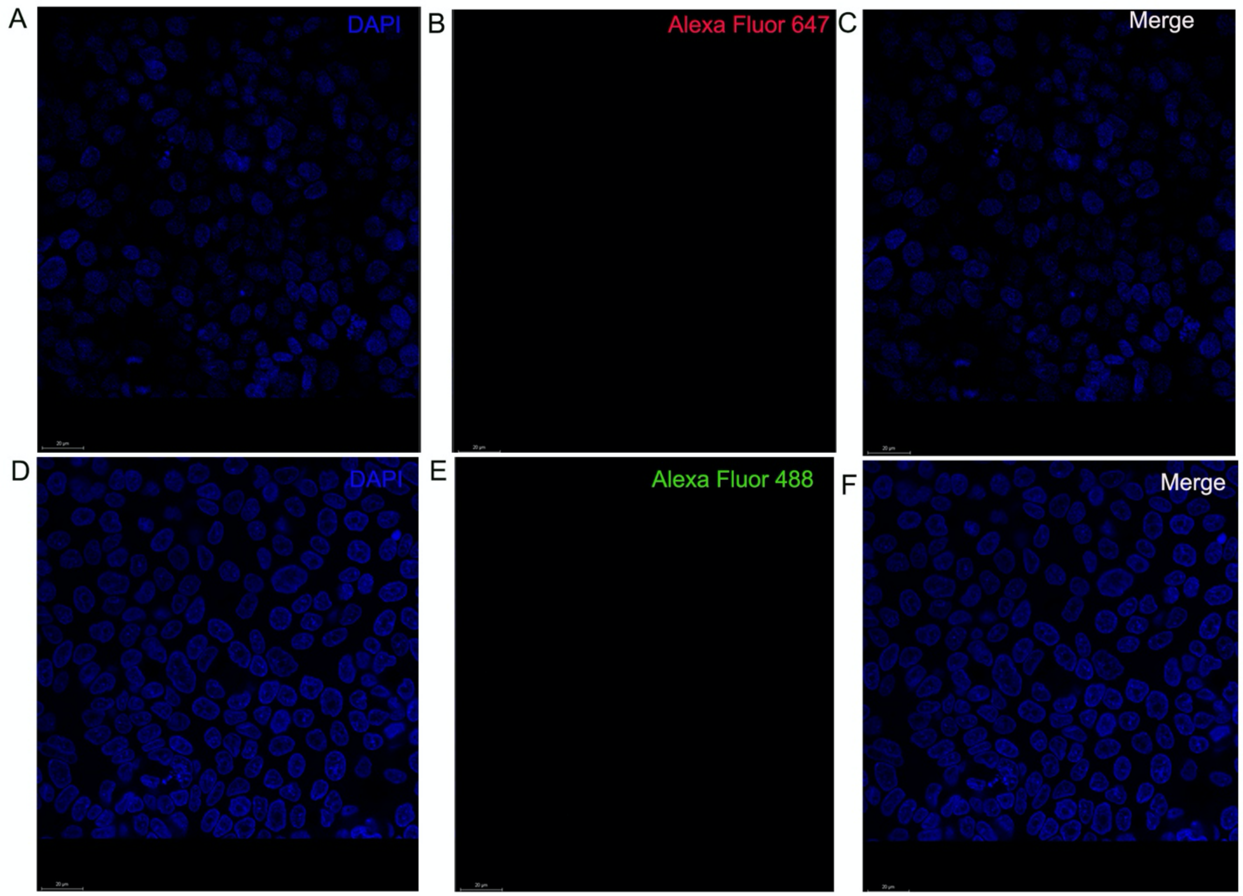

Supplementary Fig 7. Secondary antibody controls. (A-C) represent secondary antibody controls for Alexa Fluor 647. (A) nucleus labeled with DAPI (blue). (B, C) no signals detected for Alexa Fluor 647 when added without a primary antibody. (D-F) represent secondary antibody controls for Alexa Fluor 488. (D) nucleus labeled with DAPI (blue). (E, F) no signals detected for Alexa Fluor 488 when added without primary antibody.
